# An antibiotic produced by *Pseudomonas fluorescens* CFBP2392 with antifungal activity against *Rhizoctonia solani*

**DOI:** 10.3389/fmicb.2023.1286926

**Published:** 2023-11-14

**Authors:** Nadia Riera, Danilo Davyt, Rosario Durán, Gregorio Iraola, Philippe Lemanceau, Natalia Bajsa

**Affiliations:** ^1^Laboratorio de Ecología Microbiana, Instituto de Investigaciones Biológicas Clemente Estable, Montevideo, Uruguay; ^2^Laboratorio de Genómica Microbiana, Institut Pasteur de Montevideo, Montevideo, Uruguay; ^3^Laboratorio de Química Farmacéutica, Facultad de Química, Universidad de la República, Montevideo, Uruguay; ^4^Unidad Mixta de Bioquímica y Proteómica Analíticas, Institut Pasteur de Montevideo, Instituto de Investigaciones Biológicas Clemente Estable, Montevideo, Uruguay; ^5^Agroécologie, INRAE, Institut Agro, Université de Bourgogne—Université de Bourgogne Franche-Comté, Dijon, France

**Keywords:** antifungal, *Pseudomonas fluorescens*, biocontrol, *Rhizoctonia solani*, specialized metabolites, genome

## Abstract

*Pseudomonas fluorescens* CFBP2392 has been recognized as a potential biocontrol agent due to its ability to suppress damping-off and root rot disease. This isolate has antibacterial activity *in vitro* as many other strains from the *Pseudomonas fluorescens* complex. In this work, the antibacterial and antifungal activity of the strain were explored. Dual culture assays evidenced the antifungal activity of the strain against different phytopathogens: *Alternaria* sp., *Pythium ultimun*, *Fusarium oxysporum*, and *Rhizoctonia solani*. Purification of an antifungal fraction was performed by preparative HPLC from the chemical extraction of growth media. The fraction showed altered *R. solani* growth and ultrastructure. Transmission electron microscopy revealed the purified compound *induced* hypertrophied mitochondria, membranous vesicles, and a higher number of vacuoles in *R. salani* cytoplasm. In addition, co-cultivation of *P. fluorescens* CFBP2392 with *R. solani* resulted in an enlarged and deformed cell wall. To gain genomic insights on this inhibition, the complete genome of *P. fluorescens* CFBP2392 was obtained with Oxford Nanopore technology. Different biosynthetic gene clusters (BGCs) involved in specialized metabolites production including a lokisin-like and a koreenceine-like cluster were identified. In accordance with the putative BGCs identified, sequence phylogeny analysis of the MacB transporter in the lokisin-like cluster further supports the similarity with other transporters from the amphisin family. Our results give insights into the cellular effects of the purified microbial metabolite in *R. solani* ultrastructure and provide a genomic background to further explore the specialized metabolite potential.

## 1. Introduction

Molecular communication among plants and microbes is a key factor establishing both beneficial and pathogenic relationships in the rhizosphere (Stringlis et al., 2018). Bioactive compounds are part of the complex communication that shapes the microbiome of the plant, now known to have a significant role in plant’s health (Mendes et al., 2011; Turner et al., 2013; Schlaeppi and Bulgarelli, 2015; Vandenkoornhuyse et al., 2015; Zhang et al., 2017).

Fluorescent pseudomonas are ubiquitous in many environments including the rhizosphere. Many strains within this group are plant growth promoting bacteria and biocontrol agents (Haas and Défago, 2005; Weller, 2007; Höfte and Altier, 2010). Some of their positive traits include the utilization of plant exudates as a nutrient source, the production of lytic enzymes, their ability to efficiently colonize and multiply in the rhizosphere, activate the systemic immune resistance of the plant and their great genomic potential to produce bioactive small molecules (Walsh et al., 2001; Raaijmakers et al., 2002; Haas and Défago, 2005; Weller, 2007; Stringlis et al., 2018). In fact, the production of secondary metabolites from *Pseudomonas* spp. is closely linked to their ability as biocontrol agents (Thomashow et al., 1990; Walsh et al., 2001). Within the beneficial pseudomonas many are able to produce siderophores, growth promoting substances, volatile organic compounds (VOCs) as well as antibiotics. The latter include: 2,4-DAPG, derivatives of phenazine, pyrrolnitrine, pioluteorin, cyclic lipopeptides (CLP), and cell surface molecules with biosurfactant and antifungal activity (Haas and Défago, 2005).

*Pseudomonas fluorescens* A6, registered in the CIRM-CFBP French Collection for Plant Associated Bacteria^1^ as CFBP2392, was originally isolated from the rhizosphere of bean cultivated in a soil showing a low incidence of damping-off (Lemanceau and Samson, 1983) bean rhizosphere. This strain was showed to be a good root colonizer (Gamalero et al., 2004), to promote plant growth and root architecture (Gamalero et al., 2002) and to suppress *R. solani* can suppress damping-off and root rot (Berta et al., 2005). In the present work, we sought to characterize the antimicrobial spectra of the strain against phytopathogenic fungi and bacteria. We observed a strong antifungal activity against *R. solani* in dual culture assays. To further understand the mechanism of inhibition, we purified the active compound and performed dual culture assays testing the effect of the pure compound and the strain growing together with *R. solani*. Using Transmission Electron Microscopy (TEM), a structural effect in *R. solani* morphology was observed when grown in close proximity of the bacteria and the purified compound.

In recent years, the rapid development of new sequence technologies has revolutionized microbiology. An emerging field from this revolution is the discovery of new drugs using genome mining from the new pool of high-quality genomes available (Baltz, 2021). In this work, we used Oxford Nanopore Sequencing, a third-generation sequencing technology based on long reads, to obtain the complete high-quality genome of *P. fluorescens* CFBP2392. We identified different Biosynthetic Gene Clusters (BGCs) including four non-ribosomal peptide synthases (NRPS) clusters. Furthermore, we used the amino acid sequence of the ABC transporter MacB to predict the chemical nature of the metabolite exported using sequence phylogeny as previously reported (Girard et al., 2022). In agreement with the BGCs prediction an amphisin-transporter was found in this strain and other phylogenetically similar *P. fluorescens.* The lokisin-like cluster shares 85% similarity with other lokisins, predicting some structural dissimilarities with other amphisins reported thus far. Our results give relevant structural mechanistic properties of the novel antibiotic compound and provide a genomic background of the putative chemical nature.

## 2. Materials and methods

### 2.1. Antifungal activity

Antifungal activity was tested in dual culture petri dishes by inoculating mycelia in the center of the dish and a bacterial strike 4 cm on each side as previously described (Geels and Schippers, 1983). Strain *P. fluorescens* CFBP2392 was tested against *Pythium debaryanum*, *Fusarium oxysporum*, *Aspergillus niger*, *Alternaria* sp., and different isolates of *Rhizoctonia solani* (Supplementary Table 1). All assays were performed in rich (PSFM) and minimal (MMP) media. In each case, a control plate with mycelium alone was incorporated as a control and all plates were incubated at 25°C.

### 2.2. Media

Pseudomonas Agar F Modified is a modified version of Bacto Pseudomonas Agar F (Difco®) containing per liter: Tryptone 10 g, Peptone protease 10 g, K_2_HPO_4_ 1.5 g, MgSO_4_.7H_2_O 1.5 g, glycerol 15 mL, Agar 20 g, and FeCl_3_ 50 μM. MMP media was done as a modified version of Minimal media for *Pseudomonas* (Loper, 1988) containing per liter: K_2_HPO_4_ 3 g, NaH_2_PO_4_ 1 g, NH_4_Cl 1 g, MgSO_4_.7H_2_O 1.5 g, FeCl_3_ 50 μM, and glycerol 15 mL.

### 2.3. Purification of antimicrobial compounds

Strain *P. fluorescens* CFBP2392 was grown in 50 mL of PSFM medium for 3 days. Four 6-L flasks containing 2 L of PSFM were inoculated with 30 mL of the previous cultures and incubated for 3 days. The 8 L of culture were acidified with TFA to pH 1 and extracted with ethyl acetate. To separate the phases, the emulsion formed was filtered through celite (diatom skeletons). The organic phase was evaporated to dryness and the residue obtained was stored at −20°C.

### 2.4. Sample preparation for microscopy

Samples of young *R. solani* mycelium were taken from bioassays by cutting pieces of agar from the outer edge of the colony, adjacent to the bacteria or the paper disk. Fixation with 2% glutaraldehyde and 200 mM CaCl_2_ in cacodylate buffer was performed overnight at 4°C. After four washes with cacodylate buffer, half of the samples from each treatment were post-fixed with osmium tetroxide in the same buffer for 1 h at 4°C. All the samples were dehydrated using increasing concentrations of ethanol and then the inclusion process was carried out, ensuring that the apices of the hyphae were positioned in the anterior part of the blocks. The samples with double fixation were included in Epon resin and the rest in LR White resin. Semi-thin sections (0.5 μm) were made using a Reicher Ultracut microtome and a glass blade, which were stained with toluidine blue and observed under an optical microscope. Subsequently, ultrathin sections (97 nm) were cut using a 1.2 mm wide diamond blade (Diatome), which were placed on copper or gold grids pre-coated with carbon. All cuts were made with the precaution of sampling the apical zone of the mycelium.

### 2.5. Transmission electron microscopy

Observations were made using a Hitachi 7500 transmission electron microscope at 80 kV, located at INRA (Dijon, France). For each treatment and with each of the revealed ones, two grids (from two different blocks) were analyzed and approximately 15 hyphal sections were examined in each of them.

### 2.6. Measurement of cell wall thickness

Cell wall thickness measurements were made on digital images obtained by DC staining, using the Image-Pro Plus V 4.5.0.19 program (Cellular Biology Laboratory, IIBCE). Approximately 20 measurements per image were carried out and the wall thickness in nanometers was calculated from the average value (correcting for the magnification used in each image). 25–30 cells per treatment, found in two grids from two different blocks, were analyzed. The data were analyzed by ANOVA after a log10 transformation to homogenize the variances. The means were compared with Duncan’s test with *p* < 0.05.

### 2.7. DNA extraction and sequencing

A 2 mL overnight liquid culture from a single colony was used for nucleic acid extraction. DNA was extracted using PureLink® Genomic DNA Kit following the manual for Gram-negative bacteria cell lysis. Extracted DNA was fragmented to a 10 kb size using Covaris g-TUBE. Final DNA concentration was quantified fluorometrically using the Qubit 3.0 fluorimeter (Thermo Fisher Scientific). Fragmented DNA was further used to prepare the library following the Oxford Nanopore protocol Ligation sequencing gDNA (SQK-LSK109). Briefly, for DNA repair and end-prep, 500 ng fragmented DNA was treated with NEBNext FFPE DNA Repair (New England Biolabs) followed by Ultra II End Repair (New England Biolabs) in a Bio-Rad T100TM thermal cycler. Oxford Nanopore Adapter Mix (AMX) was ligated to the end-repaired adenylated DNA using Ligation Buffer (Oxford Nanopore Technologies, United Kingdom) and NEBNext Quick T4 DNA ligase at room temperature. In each step, DNA was purified using AMPure XP Reagent beads and quantified with a Qubit 2.0 Fluorometer. The library was loaded in a Flongle flow cell on a MinION Mk1C device and sequenced for 24 h.

### 2.8. Genome assembly and annotation

Sequencing finalized with a total of 779,479 reads that passed quality threshold. High accuracy basecalling and trimming was performed with guppy (V4.0.11). Processed reads were assembled with flye (V2.9) and the final assembly resulted in a genome total length of 6,645,616 bases, in one fragment, and an estimated mean coverage of 111 with a minimum overlap of 5,000. The genome was annotated with prodigal (V2.6.3).

### 2.9. Comparative genome analysis and specialized metabolites prediction

For the comparative analysis, genomes from *Pseudomonas fluorescens* group (taxon ID = 294) were downloaded from NCBI database with the—assembly-level complete flag. FastANI was used to compare *P. fluorescens* CFBP2392 with the rest, those genomes with ANI > 97% were selected for further studies (*n* = 13). Assembled genomes were annotated with prokka (v1.14.6), and pangenome estimation was analyzed with Pewit.^2^ Maximum likelihood phylogenetic analysis was performed using Pagoo library in R with a core level of 95%, resulting in 4,560 core genes (Ferrés and Iraola, 2021). Prediction of the putative antimicrobial biosynthesis clusters was performed using antiSMASH software (version 7.0; Blin et al., 2023).

### 2.10. Phylogeny of PleB protein

Pseudomonas lipopeptide export B (PleB) protein sequences were downloaded from NCBI database from each representative lipopeptide family factin, bananamide, viscosin, orfamide, poaeamide, amphisin, gacamide, putisolvin, xantholysin, entolysin, tolaasin, and peptin. Accession numbers were as follows AHZ34236.1, SDT21849.1, ESW57247.1, AGL83961.1, AGE25482.1, MBY8934714.1, ABA73958.1, ABW17379.1, AGM14936.1, CAK15812.1, PKA77400.1, and AKF46139.1, respectively. PleB sequence from region 5 of CFBP2392 was included in the analysis. We further compared seven additional PleB proteins from amphisin family from NCBI namely: ABW17379.1, MBY8934714.1, WP_064118557.1, BAF40423.1, QDF82252.1, QNL34620.1, and WP_175554031.1 together with *P. fluorescens* CFBP2392 PleB MacB_R5_CFBP2392. In all cases, sequences were aligned according to their protein sequence using PROMALS3D with standard parameters (mafft; Pei et al., 2008). The resulting alignment was used to generate the tree with iqtree (2.1.4-beta).

## 3. Results

### 3.1. Antifungal and antibacterial activity of *Pseudomonas fluorescens* CFBP2392

**Figure 1:**
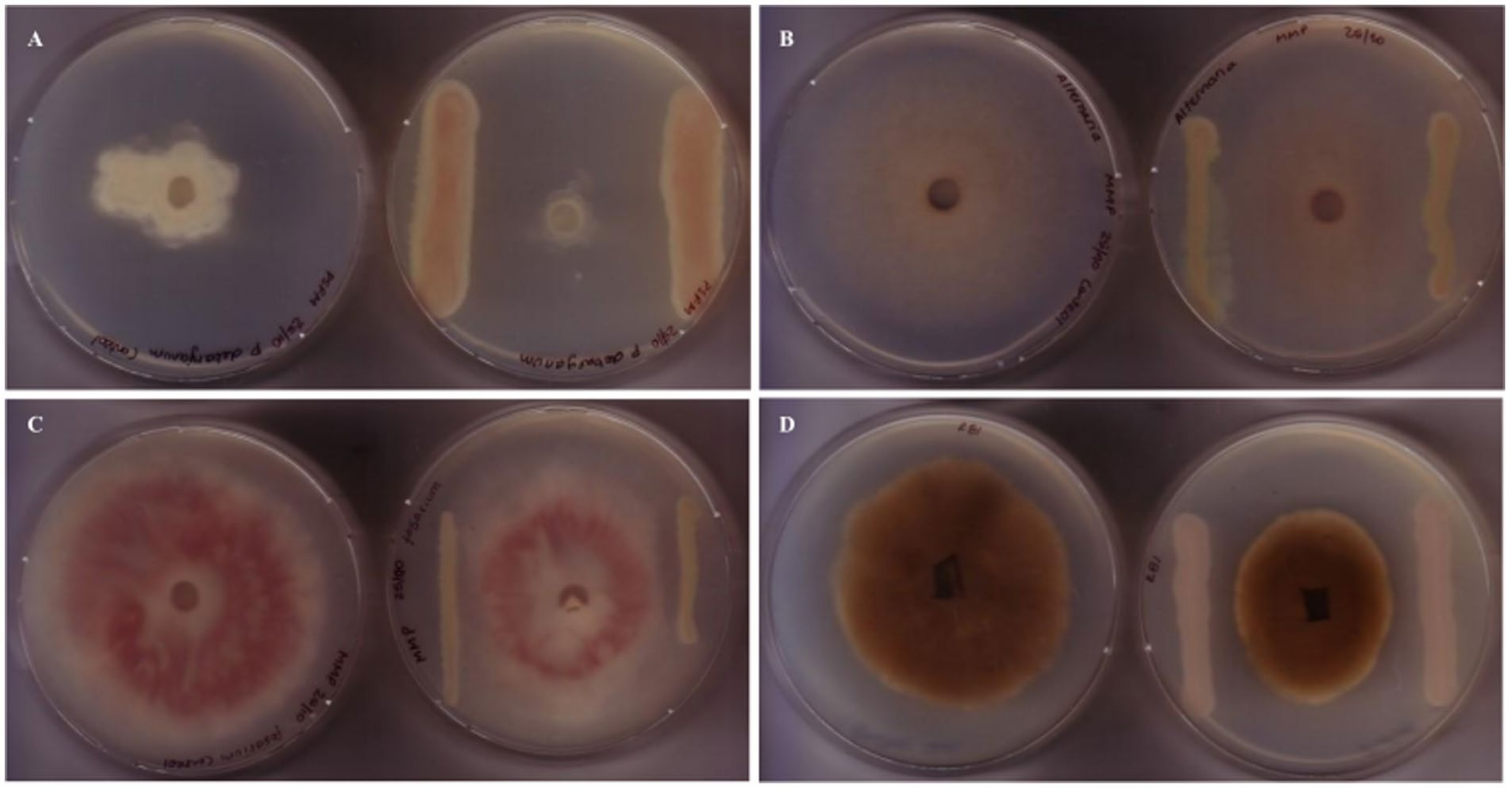
Antifungal activity of *Pseudomonas fluorescens* CFBP2392 against fungal phytopathogens. Antagonist assays against **(A)** *Fusarium oxysporum*, **(B)** *Alternaria alternata*, **(C)** *Pythium debaryanum*, and **(D)** *Rhizoctonia solani*, reveal growth defects when grown in proximity to *P. fluorescens* CFBP2392. For each case, left shows control fungal growth, right shows antagonist assay with *P. fluorescens* CFBP2392.

We screened the antimicrobial spectrum of *P. fluorescens* CFBP2392 against different fungal and bacterial isolates. Initially, *in vitro* antifungal activity was tested against four different fungal species: *Alternaria* sp., *Fusarium oxysporum*, *Pythium debaryanum*, and *Rhizoctonia solani* AG3. *Pseudomonas fluorescens* CFBP2392 was able to inhibit fungal growth *in vitro* in antagonist assay against all species. We further tested the ability to inhibit different strains within the same species and we tested the antifungal activity against three additional *R. solani* Argentinian isolates: *R. solani* 118 AG4, *R. solani* R81 AG4, and *R. solani* 109. We decided to test two different growth conditions, *Pseudomonas* minimal media (MMP) and rich media Pseudomonas Agar F Modified (PSFM) to evaluate the antimicrobial activity against these phytopathogens. We observed stronger antifungal activity *in vitro* against *F. oxysporum*, *Alternaria*, and *R. solani* when microorganisms were grown in rich media (PSFM). *Pseudomonas fluorescens* CFBP2392 presented inhibitory activity against all tested fungal isolates *in vitro* (Figure 1; Table 1; Supplementary Table 1).

**Table 1:** Antifungal activity of *Pseudomonas fluorescens* CFBP2392 against *Alternaria alternata*, *Fusarium oxysporum, Pythium debaryanum*, and four strains of *Rhizoctonia solani.*

Isolate	Inhibition PMSF	Inhibition MMP
*Alternaria* sp.	++	+
*Fusarium oxysporum*	+	+
*Pythium debaryanum*	++	+
*Rhizoctonia solani AG3*	++	
*Rhizoctonia solani 118 AG4*	+	+
*Rhizoctonia solani R81 AG4*	++	+
*Rhizoctonia solani* 109	+	+

Inhibition observed in dual culture plate assay. (++) High inhibition, (+) Inhibition.

Fluorescent pseudomonas are a group of ubiquitous well known biocontrol agents. Antibiotic compounds produced by biocontrol strains help to orchestrate microbial communities and structure. In order to explore the antibacterial spectrum of *P. fluorescens* CFBP2392, we decided to evaluate the effect on Gram positive and Gram negative bacterial phytophatogens. *Pseudomonas fluorescens* CFBP2392 was able to inhibit *Clavibacter michiganensis* but no activity was observed against the Gram negative *Ralstonia solanacearum* Rs8 or *Herbaspirillum seropedicae* Z69 (Supplementary Table 2; Supplementary Figure 1; Supplementary Methods). In addition, we decided to explore the ability of the strain to produce compounds with antimicrobial activity seen in other *Pseudomonas* spp. strains. We here show that *P. fluorescens* CFBP2392 is able to produce hydrogen cyanide (HCN) and exoproteases (Supplementary Figure 2; Supplementary Methods).

### 3.2. Production and purification of antifungal compounds

Cell free supernatant (CFS) from *P. fluorescens* CFBP2392 grown in PSFM was evaluated for its antifungal activity by incorporating either (1) no CFS (Control), (2) 2.5 mL CFS, and (3) 5 mL CFS in plates where *R. solani* was later inoculated. After 4 days, the control plate with no added CFS was covered with *R. solani* mycelia while plates with 2.5 mL were only half covered with mycelia and for plates with 5 mL CFS we observed no fungal growth. We decided to do a first chemical extraction of CFS using ethyl acetate and resuspending the extract in methanol (see methods). We confirmed the organic phase had antifungal activity as compared to control with methanol in plate bioassays (Supplementary Figure 2). For further analysis, we performed the same protocol for chemical extraction from an initial culture of 8 L, and we purified the organic phase with adsorption chromatography. Fractions with activity were evaluated with a bioassay as previously described and further purified with HPLC collecting a single peak (Supplementary Figure 3). To evaluate the impact in *R. solani* mycelia, the fraction corresponding to a single peak was used in subsequent microscopic analysis.

### 3.3. *Pseudomonas fluorescens* CFBP2392 induces alterations in *Rhizoctonia solani* cell wall ultrastructure

**Figure 2:**
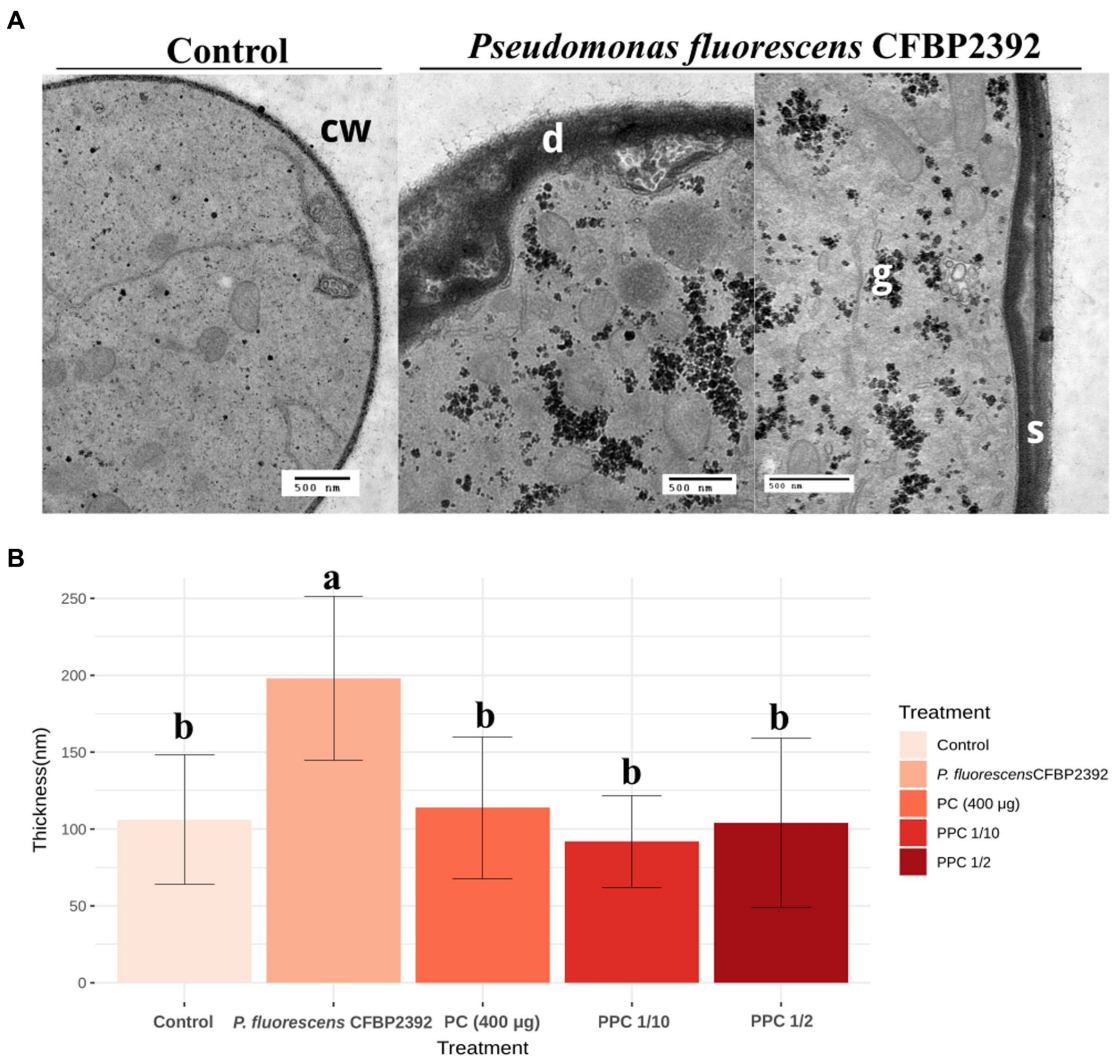
Effect of *Pseudomonas fluorescens* CFBP2392 in *Rhizoctonia solani* cell wall thickness. **(A)** *Pseudomonas fluorecsens* CFBP2392 induces deformations (d) and separation (s) of *R. solani* cell wall (cw). Glycogen accumulation is also observed (g). Images were obtained with TEM using periodic acid-silver thiocarbohydrazide-proteinate (PATAg) staining. **(B)** Cell wall thickness for *P. fluorescens*, purified compound (PC), partially purified compound (PPC) 1/2 dilution, and PPC 1/10 dilution measured in 20 points. Values represent the average thickness of 25–30 sections from two different grids. ANOVA was calculated (*n* = 135) after a log10 transformation to homogenize standard deviation.

*Rhizoctonia solani* cell wall enlargement due to antifungal exposure was previously reported (Benhamou and Chet, 1993; Bianchi et al., 1997; Benhamou et al., 1999). We sought to evaluate the modifications in the cell wall thickness in the presence of bacteria or purified compound (PC) or partially purified compound (PPC). The antifungal activity of the purified extract was tested *in vitro* and samples for microscopy were prepared from these plates (Supplementary Figure 4). A strong enlargement effect was observed when comparing *R. solani* cell wall thickness in the presence of *P. fluorescens* CFBP2392 against untreated control, but not in the presence of the purified or partially purified compound (Figure 2). The morphological alterations in cell wall morphology were clear in the presence of *P. fluorescens*. In some regions, *P. fluorescens* induced deformations in the fungal cell wall and separation toward the cell interior. These changes were not observed for the purified or partially purified compound. In light of these results, we cannot attribute the changes in cell wall morphology to the purified antibiotic. Further data are needed to explain the cause of *P. fluorescens* effect on *R. solani* ultrastructural changes.

### 3.4. *Pseudomonas fluorescens* CFBP2392 triggers cytoplasmic alterations in *Rhizoctonia solani*

**Figure 3:**
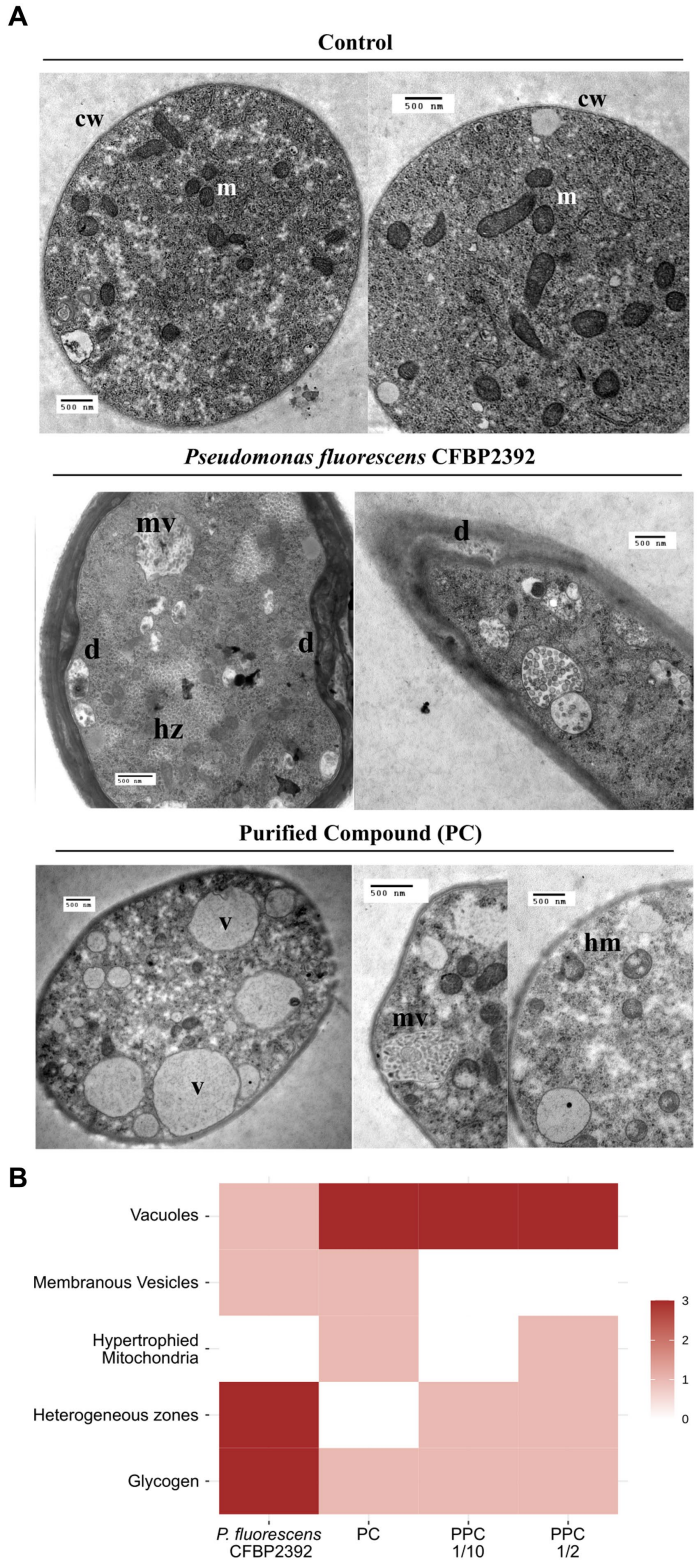
Morphological changes in *Rhizoctonia solani* observed in the presence of *Pseudomonas fluorescens* CFBP2392 or its purified compound (PC). **(A)** Images were obtained with TEM using double contrast staining. m, mitochondria; cw, cell wall; hm, hypertrophied mitochondria; mv, membranous vesicles; hz, heterogeneus zones; and v, vacuoles. **(B)** Summary of ultrastructural changes with high (red), medium (pink), or no alterations (blank) observed in *R. solani*. All changes expressed as compared to untreated *R. solani* growth.

Fungal tissue grown in the presence of *P. fluorescens* CFBP2392 or purified compound (PC) presented strong structural and morphological changes in cytoplasmic organelles as compared to untreated *R. solani* control (Figure 3A). Heterogeneous regions were abundantly present in the cell, which corresponded with glycogen accumulation (based on silver proteinate-periodic acid and thiocarbohydrazide staining). An increase in the number of vacuoles was observed in the fungal cell tissue when exposed to partially purified compound (PPC) and CP but not in the presence of *P. fluorescens* CFBP2392 (Figure 3B). In a similar way, PC also produced membranous vesicles with granular content in *R. solani*. The most significant change induced by PC and PPC in cytoplasm morphology, however, is seen in the mitochondria. The purified fraction of antifungal compound triggered changes in mitochondria morphology resulting in hypertrophy (Figure 3B).

### 3.5. Complete genomic sequencing of *Pseudomonas fluorescens* CFBP2392

**Figure 4:**
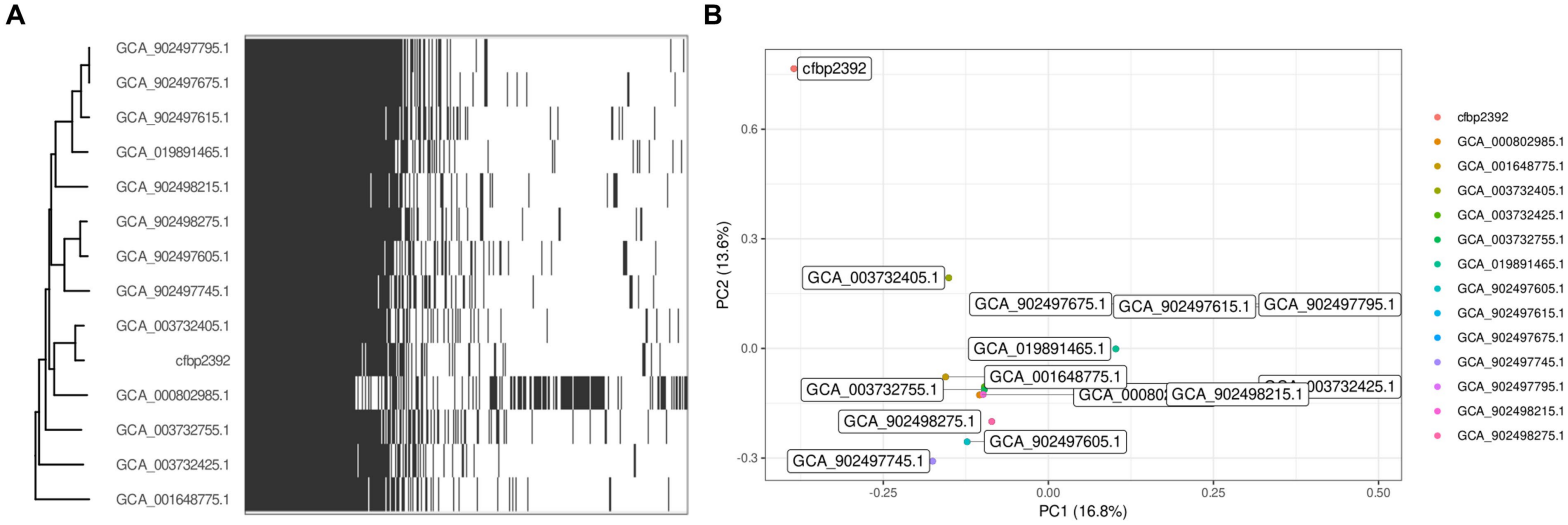
Pangenome analysis with 14 selected strains of *Pseudomonas fluorescens.* **(A)** Heatmap represents presence and absence of genes within each genome clustered by phylogeny based on predicted core genes (*n* = 4,560). **(B)** Biplot visualization of the two main components of the PCA.

We decided to explore the genomic content of the strain by obtaining the complete genome using Oxford Nanopore Sequencing Technologies. High accuracy base calling and read processing were performed with guppy (4.0.11) and the obtained reads were assembled with flye (V2.9). The resulting sequence mapped to a single circular chromosome with an estimated mean coverage of 111 and a minimum overlap of 5,000 pb (Supplementary Table 3). We speculated that other members of the *Pseudomonas fluorescens* complex community may have been previously characterized for their ability to produce specialized antibiotics. We collected all complete genomes from *Pseudomonas fluorescens* from NCBI database and performed a pangenome analysis to study the distribution of core/accessory genes among the group. We obtained 173 genomic sequences from NCBI under the taxon ID 294 and we filtered them by genome quality with CheckM, which resulted in 165 genomes. Due to the high variability among the complex, we further filtered those genomes with high ANI (>85%) with *P. fluorescence* CFBP2392 and that resulted in a smaller set of 14 genomes. We used the pangenome estimation tool Pewit to visualize the genomic content of this selected group. The core genome of these subsets was composed of 4,560 genes. Principal component analysis of *P. fluorescens* CFBP2392 accessory genome shows this strain separates from the other in the PC1 and PC2 dimensions (Figure 4).

### 3.6. Genome mining predicts four putative biosynthesis gene clusters of NRPSs and primary metabolites with potential antibiotic potential

**Figure 5:**
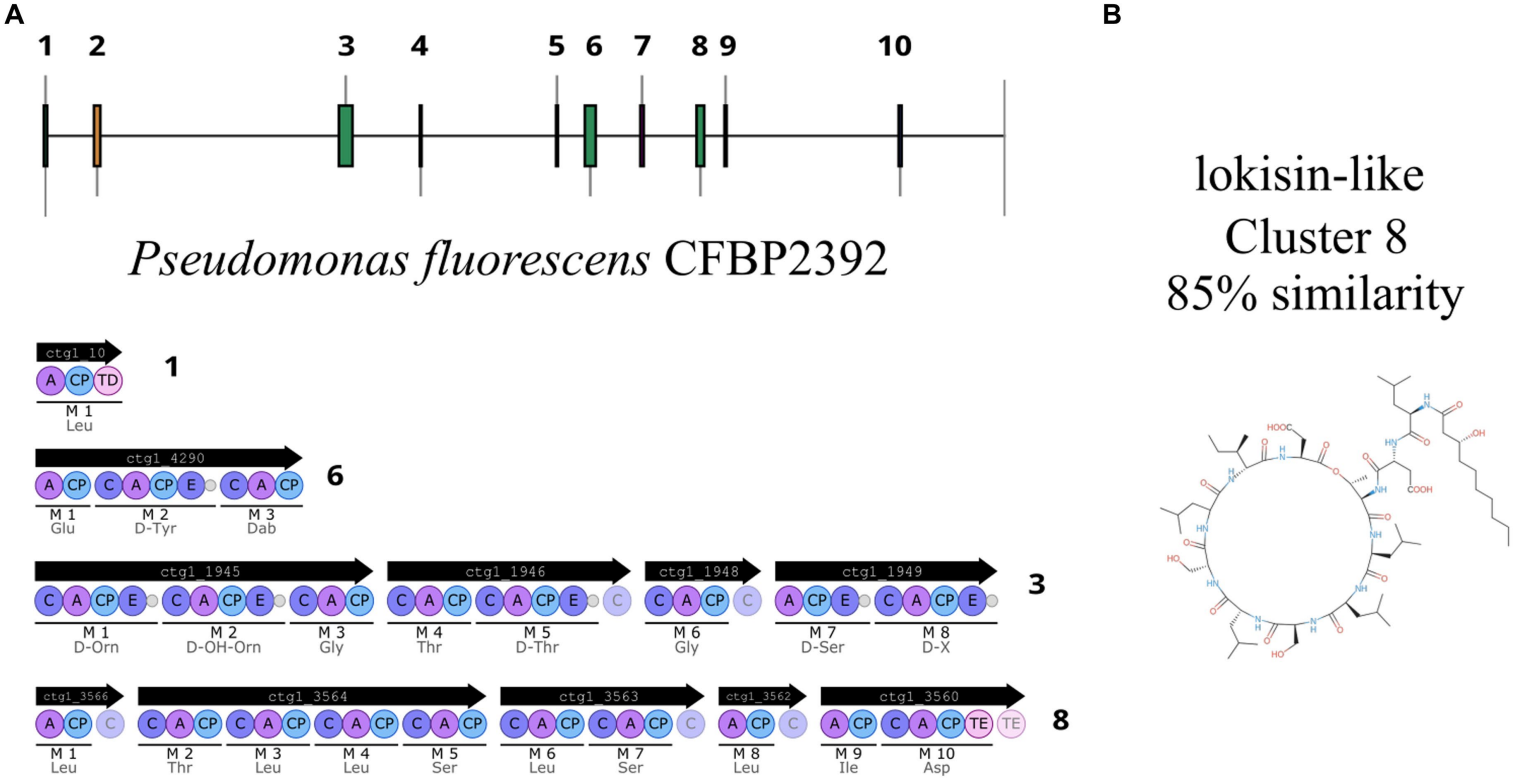
*Pseudomonas fluorescens* CFBP2392 genome analysis predicts 10 putative BGCs. **(A)** Four NRPS regions are predicted within *P. fluorescens* CFBP2392 genome. **(B)** Chemical structure of lokisin as reported in MIBiG.

In recent years, genome mining and next generation sequencing proved to be useful tools to predict putative specialized metabolites. Using antiSMASH (v7.0.0), we mined for putative biosynthetic gene clusters within the genome, as a result, 10 putative regions were predicted (Figure 5). Two of these regions were predicted to encode ribosomally synthesized and post-tranlationally modified peptides (RIPP)-like antibiotics, three NRPS and one NRPS-like, one betalactone, one siderophore, one RRE-containing, and one arylpolyene (Supplementary Table 4).

**Figure 6:**
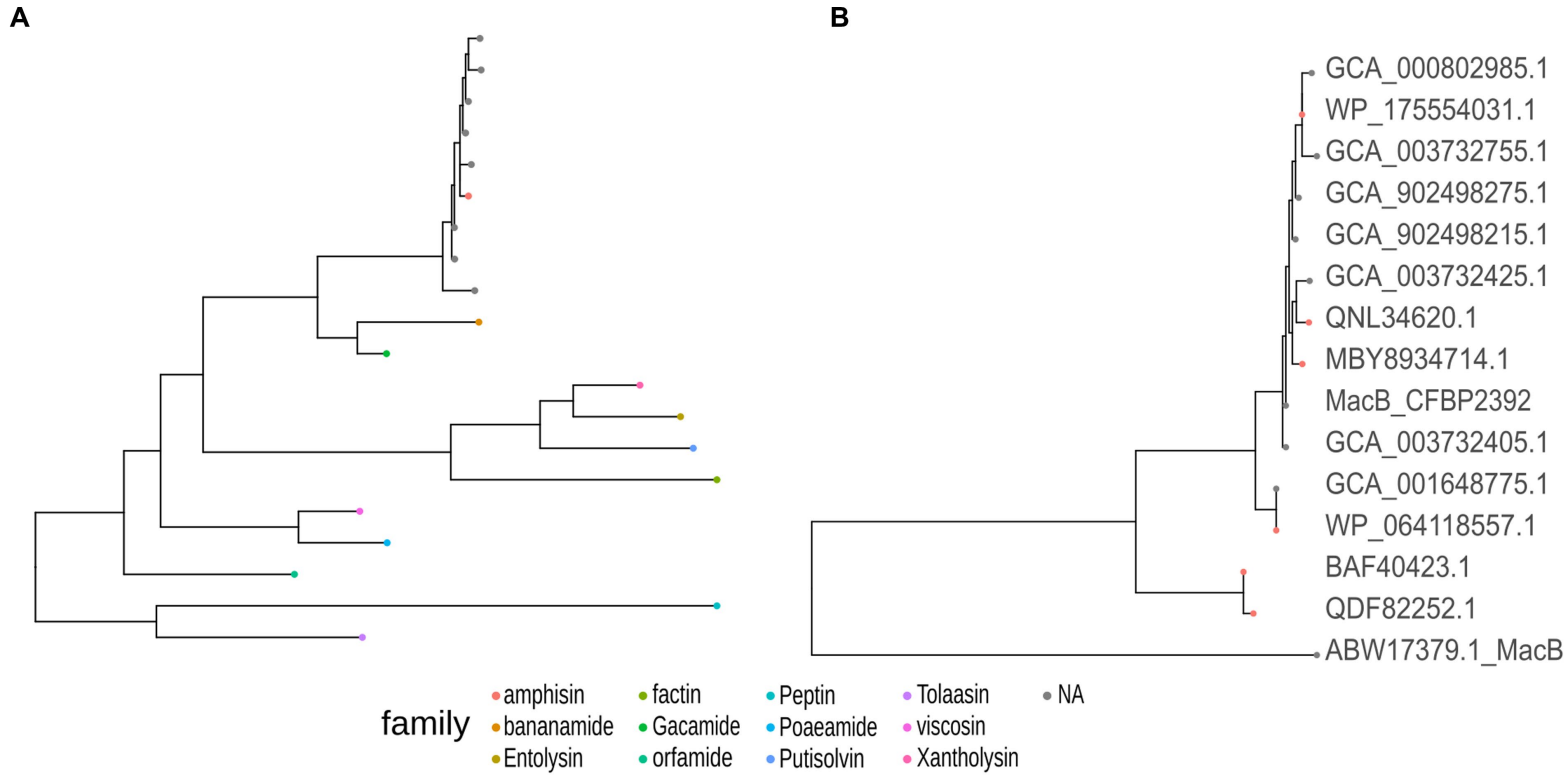
*Pseudomonas fluorescens* CFBP2392 and other genomically related *P. fluorescens* MacB amino acid sequence presents high homology with the amphisin family of Pseudomonas export lipopeptides proteins. **(A)** MacB sequences from the selected genomically related *P. fluorescens* (gray) cluster with the family amphisin of lipopeptides (red). **(B)** Amino acid sequence from six genetically or chemically characterized ABC transporters from the amphisin family (red) with nine sequences from selected *P. fluorescens* (gray).

One region shows a high percentage of identity with lokisin antibiotic (85%), the rest presented low identity to known metabolites or no predicted metabolites at all (Supplementary Figure 5). The predicted BGCs in the genome are in accordance with the other 13 genomes of *P. fluorescens* with high sequence identity to *P. fluorescens* CFBP2392 (Supplementary Figure 6). We decided to compare the amino acid sequence of *P. fluorescens* CFBP2392 MacB with other genomically related genomes together with representative sequences from genetically or chemically known MacB families. We used the MacB sequence of the other 13 strains selected for the pangenome analysis together with representative members of different lipopeptide families. Therefore, we obtained sequences from the amphisin, bananamide, entolysin, factin, gacamide, orfamide, peptin, poaeamide, putisolvin, tolaasin, viscosin, and xantholysin families and we compared them using the structure-based algorithm PROMALS3D (PROfile Multiple Alignment with predicted Local Structures and 3D constraints; Figure 6). As expected, based on MacB phylogeny, *P. fluorescens* CFBP2392 as well as the other 13 MacB sequences, clustered with amphisin family of lipopeptides with a sequence similar to *Pseudomonas fluorescens* MBY8934714.1, *P. fluorescens* WP_064118557.1, and *Peudomonas* sp. QNL34620.1 (Figure 6; Supplementary Table 5).

AntiSMASH also predicted several saccharides and fatty acids in *P. fluorescens* primary metabolites (Supplementary Table 4). Of note, the algorithm predicted a fatty acid biosynthesis region with 100% identity to koreenceine (region 4), a polyketide produced by *Pseudomonas koreencis.* Recently, this chemical analog of plants tetrahydropyridine alkaloids, was identified as responsible for the inhibition of the Gram negative bacteria *Flavobacterium johnsoniae* (Lozano et al., 2019).

## 4. Discussion

### 4.1. Antimicrobial activity

The antifungal activity of *P. fluorescens* CFBP2392 was tested against *R. solani*, *F. oxysporum*, *P. debaryanum*, and *Alternaria* sp. evaluating in each case the growth of the mycelium vs. a control without bacterial inoculum. We found inhibition of the fungal strains, consistent with previous literature of *P. fluorescens* CFBP2392 against pathogenic fungi (Bajsa et al., 2003; Berta et al., 2005). In this work, we observed greater inhibition of *R. solani* growth as compared to the other species of phytopathogenic fungi. For this reason, a second antagonism assay was carried out, testing the ability to inhibitory of *P. fluorescens* CFBP2392 against different isolates of *R. solani.* We selected four different isolates, belonging to two anastomosis groups (AG3 and AG4). It was observed that the greatest inhibition occurred with the AG3 LEM strain. Based on these results, *R. solani* was used to evaluate the morphological effect using TEM and to corroborate the production of the metabolite and its presence or absence during the purification process.

The antimicrobial activity of *P. fluorescens* CFBP2392 was observed against the Gram positive actinomycete *C. michiganensis* but not against the plant growth promoting *H. seropericae*. In a previous work from our lab, activity of the *P. fluorescens* UP61 strain was reported *in vitro* against bacteria (La Fuente et al., 2004). *Pseudomonas fluorescens* UP61 activity was screened against 12 phytopathogenic bacteria and detected against six of them: *Agrobacterium tumefaciens*, *C. michiganensis* subspecies *michiganensis*, *Corynebacterium indiosum*, *Pseudomonas syringae* pv. *atrofaciens*, *Pseudomonas syringae* pv. *syringae*, and *Xanthomonas campestris* pv. *mavacearum* (La Fuente et al., 2004).

For the antifungal and antibacterial activity presented, the inhibitory capacity of the strain was tested, not the pure antibiotic. This implies that the observed inhibition may also be due to other active compounds produced by the strain. Most of the bacteria within the *Pseudomonas* genus have more than one inhibition mechanism (Haas and Défago, 2005; Arora et al., 2008). We show that this particular strain is capable of producing pyoverdine, hydrocyanic acid (HCN), and proteases. It was taken into account that pyoverdine synthesis is inhibited in iron-rich culture media and that hydrocyanic acid is too volatile to be responsible for inhibition. For all cases in this work, culture media with added iron were used.

### 4.2. Modifications in the cell wall of *Rhizoctonia solani*

In the observed hyphae cuts, it was verified that the portions of sampled mycelium contained active cells, corresponding to the growth zone of the fungus. The presence of the bacteria or the antifungal compound did not significantly change the cellular state of *R. solani*, which was estimated by evaluating the presence of symptoms of mycelial aging (less electron dense cytosol, greater presence of vacuoles, and less abundance of organelles such as mitochondria and ribosomes). Alterations in the cell wall of the fungus produced by *P. fluorescens* CFBP2392 were also observed during the interaction of these two microorganisms on tomato roots (Lherminier et al., 1999). A thickening of the wall of *R. solani* that doubled the width with respect to the control was reported when the fungus was treated with a garlic plant extract (Bianchi et al., 1997) and after exposure to plant chitinases (Benhamou et al., 1999). In contrast with our observations, the antibiotic phenazine 1 carboxamide was reported to produce thinning and deformations in *R. solani* cell wall (Xiang et al., 2018). Deformations similar to those produced by *P. fluorescens* CFBP2392 were observed during the interaction of *R. solani* with the mycoparasite *Pythium oligandrum* (Benhamou et al., 1999).

In *Mucor mucedo* (Zygomycete), a thickening of the cell wall was also observed induced by the fungicides pentachloronitrobenzene (PCNB) and terrazole, although in the latter case it appears to be a non-specific effect reflecting damage to mitochondria (Casperson and Lyr, 1975; Lyr et al., 1977). However, in studies carried out with *Phytophthora cactorum*, PCNB did not cause any deformation of the wall (Casperson and Lyr, 1975). Cell wall distortions could not be attributed to the action of the compound purified from *P. fluorescens* CFBP2392 and thus could be possibly related to the production of other antifungal factors by the bacteria such as hydrolytic exoenzymes. In this work, we report that the strain is able to produce exoproteases and hydrocyanic acid, and it had previously been reported that produces lipases and chitinases (Andreucci et al., 1999). Similarly, *Paenibacillus polymyxa* NMA1017 and *Burkholderia cenocepacea* CACua-24 strains were reported to degrade *R. solani* cell wall as observed by Scanning Electron Microscopy (SEM; Chávez-Ramírez et al., 2020). In other *Pseudomonas* spp. strains, the importance of hydrolytic exoenzymes in their antagonistic activities was demonstrated. *Pseudomonas fluorescens* BL915 strain inhibited damping off caused by *R. solani* to the production of chitinolytic enzymes among other antifungal factors (Gaffney et al., 1994). The production of chitinases and β-1,3 glucanases to digest fungal cell wall had been also reported (Gooday, 1990; Leah et al., 1991; Fridlender et al., 1993).

### 4.3. Cytoplasmic alterations in *Rhizoctonia solani*

After treatment with *P. fluorescens* CFBP2392, and to a lesser degree with partially purified compound, an increase in glycogen accumulation was observed in the cytosol of *R. solani*. In a study of the effect of pentachloronitrobenzene (PCNB) on the ultrastructure of *Mucor mucedo* and *Phytophthora cactorum*, an increase in vacuole formation was also observed (Casperson and Lyr, 1975). Another effect observed in this work was the appearance of membranous vesicles with unknown granular content. The most noticeable action produced by the purified antibiotic was on the appearance of the mitochondria of *R. solani*. These were observed larger than in the control and with their disorganized membranes.

Similar to the effect caused by the purified metabolite from *P. fluorescens* CFBP2392 on mitochondria is the one caused by that produced by cereulide, a cyclic dodecadepsipeptide produced by *Bacillus cereus* strains. This toxin causes loss of motility in pig spermatozoa and an increase in the size of their mitochondria, but without depleting cellular ATP (allows phosphorylation at the substrate level) or damaging the plasma membrane. These symptoms are similar to those produced by the ionophores valinomycin and gramicidin, indicating that cereulide acts as a channel-forming ionophore, damaging mitochondria, and blocking oxidative phosphorylation in these organelles, necessary for sperm motility (Andersson et al., 1998). TEM observations of the purified antibiotic phenazine 1 carboxamide in *R. solani* cells show twisted septum, organelles difficult to discern, and mitochondria disappear (Xiang et al., 2018). In the study of Xiang and coworkers, gene expression of complex I of the mitochondria electron transporter chain and chitin synthase was decreased in the presence of phenazine 1 carboxamide, consistent with the observed effect on both the mitochondria and the cell wall.

Two fungal strains, *Alternaria longipes* and *Epicoccum nigrum*, had also been reported to inhibit *R. solani* and evoke an effect in the cell wall (Lahlali and Hijri, 2010). In addition, there are two fungicides PCNB and terrazole whose main effect is on mitochondria, as observed by TEM. PCNB causes diffuse lysis of the internal structure of these organelles in *Mucor mucedo* and *Phytophthora cactorum*, and also an increase in the perinuclear space (Casperson and Lyr, 1975). In contrast, terrazole attacks the inner membrane of *Mucor mucedo* mitochondria leading to complete lysis of said organelles. Other affected membranes are the plasma membrane, which elongates and invaginates from the cell wall but remains intact, and the nuclear envelope, which shows vesicles between the membranes (Casperson and Lyr, 1975). The observed effects are induced by a release of phospholipases within mitochondria, and perhaps other membranes. The decrease in phosphorylation activity in mitochondria had as a side effect a pathological thickening of the cell wall (Lyr et al., 1977).

### 4.4. Genome mining and specialized metabolite prediction

In the recent years, the development of new sequencing technologies together with the emergence of new and improved genome mining techniques has revolutionized the search of new natural compounds (Ziemert et al., 2016; Albarano et al., 2020; Baltz, 2021; Blin et al., 2023). The high quality finished-genomes obtained with long-read sequencing third generation techniques such as Oxford Nanopore highly improves the prediction of biosynthetic gene cluster (BGCs). We found that *P. fluorescens* CFBP2392 harbors different BGCs clusters across its genome. In particular, we identified a lokisin-like NRPS and a koreenceine-like polyketide cluster that were previously reported to have antimicrobial activity (Lozano et al., 2019; Omoboye et al., 2019).

Lokisin is a well described lipopeptide with plant growth promoting activities. Lokisin can induce systemic resistance against rice blast disease (Omoboye et al., 2019), reduce *P. ultimun* infection in tomato (Hultberg et al., 2010), and has reported antifungal activity (Gu et al., 2020). This lipopeptide is part of the amphisin family as classified according to the total sequence length of the peptide and the size of the macrocycle (Girard et al., 2022). Lipopeptides are transported from the periplasm to the outer cell compartment by a tripartite efflux pump formed with a MacB ABC transporter, a MacA adaptor protein in the periplasm, and a TolC outer membrane protein that exports the molecule outside the cell in Gram negative bacteria (Greene et al., 2018). MacB, recently proposed to be renamed PleB (Girard et al., 2022), is conserved in the ATP-binding domain and the integral membrane helical domain but varies significantly in the domain binding the lipopeptide. Recently, due to the high variability in the substrate binding site, PleB phylogeny has been proposed as a good indicator of the chemical nature of lipopeptides exported out of the cell (Girard et al., 2022). In accordance with the genetic similarity found in the lokisin-cluster by antiSMASH, we found that PleB indeed clustered with the amphisin family suggesting a similar binding substrate as those previously reported.

## 5. Conclusion

The strain *Pseudomonas fluorescens* CFBP2392 was previously proposed as a promising biocontrol strain. Here we report the antifungal activity of the strain against *F. oxysporum*, *P. debaryanum*, *Alternaria* sp., and four *R. solani*, isolates. In addition, we observed antimicrobial activity against the Gram positive phyhtopathogen *C. michiganensis in vitro.* We purified an active compound from *P. fluorescens* CFBP2392 and evaluated its effect by TEM against *R. solani*. We observed that the strain and the purified chemical compound could produce morphological changes in *R. solani* morphology. Dual culture assay with the strain produced a thickening of the fungal cell wall. The purified compound produced alterations in the organelles including hypertrophy in mitochondria, membranous vesicles and a larger number of vacuoles per cell. The complete genome sequence of *P. fluorescens* CFBP2392 was sequenced using long-read technology Oxford Nanopore sequencing. We explore genomes from other members of the *Pseudomonas fluorescens* complex in comparison with that of *P. fluorescens* CFBP2392. The profile of BGCs of *P. fluoresces* CFBP2392 was similar to that of other members of the complex with high ANI, all of them showing a lokisin-like cluster. The lokisin-like cluster from *P. fluorescens* CFBP2392 predicts a cyclic decapeptide NRPS with a MacAB transport system. Based on antiSMASH prediction as well as MacB phylogeny the predicted metabolite seems to follow in the amphisin family of cyclopeptides with previously reported plant growth-promoting properties. We further identified a koreenceine-like cluster that could potentially explain some of the antimicrobial activity. Our results give new insights in *P. fluorescens* CFBP2392 antimicrobial activity and provide a genomic background to explore its metabolic potential. Further experiments are needed to elucidate the chemical structure of the antifungal compound.

## Data availability statement

The datasets presented in this study can be found in online repositories. The names of the repository/repositories and accession number(s) can be found in the article/Supplementary material.

## Author contributions

NR: Conceptualization, Data curation, Investigation, Methodology, Visualization, Writing – original draft. DD: Conceptualization, Resources, Writing – review & editing. RD: Conceptualization, Resources, Writing – review & editing. GI: Conceptualization, Resources, Writing – review & editing. PL: Conceptualization, Resources, Writing – review & editing, Methodology. NB: Conceptualization, Methodology, Data curation, Funding acquisition, Investigation, Project administration, Visualization, Writing – original draft.

## Footnotes

^1^ https://cirm-cfbp.fr/

^2^ https://github.com/iferres/pewit
