# Supplementary material for: An antibiotic produced by Pseudomonas fluorescens CFBP2392 with antifungal activity against Rhizoctonia solani
Source: Front Microbiol. 2023 Nov 14;14:1286926. doi: 10.3389/fmicb.2023.1286926 (PMC10682437; doi:10.3389/fmicb.2023.1286926)
Supplement: Supplementary file 1 [file Data_Sheet_1.PDF]

# Supplementary Material

## 1 Supplementary Methods

### 1.1 Antibacterial activity

Antibacterial activity was tested by inoculating *P. fluorescens* CFBP2392 on PSFM plates on glass petri dishes and incubated overnight at 25 ºC. The following day plates were subjected to chloroform vapor over a period of one hour to dill the bacteria and were left in the fume hood until all vapors were fully evaporated. A liquid culture of the tested bacteria (*Clavibacter michiganensis* LEM and *C. michiganensis* R33/2, *Ralstonia solanacearum* Rs8 and *Herbaspirillum seropedicae* Z69) was prepared in NB media overnight in a shaker at 200 rpm and 30 ºC. A second layer of soft agar medium (0.6%) with an incorporated culture of 102 cfu/mL was set on the top. The second layer was incubated at 25 ºC overnight. Positive inhibition was recorded when there was the presence of an inhibitory halo. Each assay was repeated per triplicate and plates wer incubated at 25 ºC for 24 hours. Positive antimicrobial activity was reported when the presence of an inhibitory halo was observed.

### 1.2 HCN production

The bacteria were grown in tubes containing 5 ml of KB medium for 24h. 10 µl of this culture were seeded on a solid medium containing 100 g/L of skim milk powder, 1.5 g/L yeast extract and 15 g/L agar. Activity proteolysis is visualized by the appearance of a transparent halo around the bacterial inoculum (Dunn et al., 1997).

### 1.3 Exoprotease detection

Bacteria were streaked on glycine-supplemented KB plates (4.4 g/L) and FeCl3 (100 µM) and allowed to grow for 24 h at 30 oC. Then it was placed on the lid of the plates a disc of filter paper impregnated with picric acid (0.5%) and Na2CO3 (2%), they were sealed with Parafilm® and incubated at 25 oC. The color change of the paper from yellow to red indicates HCN production (Bakker and Schippers, 1987).

### 1.4 HPLC: high pressure liquid chromatography

A preparative C8 reverse phase column (Aquapore Octyl 20 µm; BownleeTM Applied Biosystems) was used. The samples were injected dissolved in 25% acetonitrile-0.1% TFA (800 µl for the extract corresponding to 10 mL of culture), after centrifuging them at 11270 *g* for 5 min to eliminate the particles. LKB Bromma brand equipment was used: two model 2150 pumps and a model 2252 controller, located in the Analytical Biochemistry Laboratory of the Instituto de Investigaciones Biológicas Clemente Estable (Montevideo, Uruguay). The mobile phase consisted of a linear gradient of acetonitrile-TFA 0.1% in the following steps: 0-2% from 0 to 10 min, 2-12% from 10 to 30 min, 12-42% from 30 to 60 min, and 42 -90% from 60 to 75 min, with a flow of 2 mL/min. Detection was performed by absorption at 250 nm in a cell with 6.5 mm optical path length, using a recorder with a

paper speed of 2 mm/min. The solvent of the collected fractions was evaporated and later the samples were lyophilized.

## 1.5 Bioassays for microscopy

Young *R. solani* RB1 mycelium, previously grown for four days in the same medium, was placed in the center of the PSFM plates. For direct bacterial antagonism the *P. fluorescens* CFBP2392 strain was inoculated in three areas of the plate, 2.5 cm from the fungus. In addition, antifungal compounds produced by *P. fluorescens* CFBP2392 were plated on filter paper discs using the same design. 400 µg per disk of the purified compound (CP) or 25 µl of the 1/10 or 1/2 dilutions of the partially purified compound (CPP) were used. As a control, paper discs with water (solvent for PC) or with acetone (solvent for CPP) were used. All treatments were performed in triplicate. The plates were incubated for three days at 25°C.

## 1.6 Co-occurrence analysis of BGCs

# 2 Supplementary Tables

For more information on Supplementary Material and for details on the different file types accepted, please see here.

**Supplementary Table 1.** Fungal isolates used in this study

| Isolate | Country | Source | Characteristics |
|---|---|---|---|
| *Alternaria* sp. | Uruguay | Departamento de Biología Molecular, IIBCE | Phytopathogenic isolate |
| *Fusarium oxysporum* | United States | Root Disease and Biological Control Unit, USDA | Phytopathogenic isolate from pea (arveja) |
| *Pythium debaryanum* | Uruguay | Sección Protección Vegetal INIA, Las Brujas | Phytopathogenic isolate |
| *Rhizoctonia solani AG3* | United States | Department of Plant Pathology, Minnessotta | Phytopathogenic isolate |
| *Rhizoctonia solani 118 AG4* | Argentina | Instituto de Microbiología y Zoología | Phytopathogenic isolate |

| | | | |
|---|---|---|---|
| | | Agrícola. INTA Castelar | |
| *Rhizoctonia solani R81 AG4* | Argentina | Instituto de Microbiología y Zoología Agrícola. INTA Castelar | Phytopathogenic isolate |
| *Rhizoctonia solani* 109 | Argentina | Instituto de Microbiología y Zoología Agrícola. INTA Castelar | Phytopathogenic isolate |

**Supplementary Table 2.** Bacterial isolates used in this study

| Isolate | Country | Source | Characteristics |
|---|---|---|---|
| *Clavibacter michiganensis* LEM | Uruguay | Laboratorio de Ecología Microbiana, IIBCE | Isolated from tomato |
| | | | |
| *Ralstonia solanacearum* Rs8 | Uruguay | Departamento de Biología Molecular, IIBCE | Pathogenic, infects potato |
| *Herbaspirillum seropedicae* Z69 | Brazil | Baldani et al 1986 | Nitrogen fixing bacterium, isolated from rice rhizosphere |

**Supplementary Table 3.** Assembly statistics of *P. fluorescens* CFBP2392

| **Assembly statistics** | |
|---|---|
| Total length: | 6656414 |
| Fragments: | 1 |
| Fragments N50: | 6656414 |
| Largest frg: | 6656414 |
| Scaffolds: | 0 |
| Mean coverage: | 111 |

**Supplementary Table 4.** AntiSMASH predicted regions for *P. fluorescens* CFBP2392

| AntiSMASH relaxed (primary metabolites) | | | | | | |
|---|---|---|---|---|---|---|
| **Region** | **Type** | **From** | **To** | **Most similar known cluster** | | **Similarity** |
| Region 1 | NRPS-like | 1,822 | 24,473 | fragin | NRP | 0,37 |
| Region 2 | arylpolyene,halogenated | 348,591 | 392,195 | APE Vf | Other | 0,4 |
| Region 3 | saccharide | 420,589 | 464,709 | lipopolysaccharide | Saccharide | 0,36 |
| Region 4 | fatty_acid | 720,696 | 744,651 | koreenceine A/koreenceine B/koreenceine C/koreenceine D | Polyketide | 1 |
| Region 5 | saccharide | 1,489,662 | 1,513,578 | | | |
| Region 6 | fatty_acid | 1,541,419 | 1,561,479 | | | |
| Region 7 | saccharide,fatty_acid | 1,658,541 | 1,698,084 | pseudaminic acid | Other | 0,22 |
| Region 8 | NRP-metallophore, NRPS | 2,043,530 | 2,137,270 | Pf-5 pyoverdine | NRP | 0,2 |
| Region 9 | saccharide | 2,319,899 | 2,350,559 | polysaccharide B | Saccharide | 0,06 |
| Region 10 | saccharide,NI-siderophore | 2,578,532 | 2,615,845 | gladiostatin A | NRP+Polyketide | 0,17 |
| Region 11 | saccharide | 3,219,007 | 3,243,219 | | | |
| Region 12 | saccharide | 3,274,842 | 3,294,754 | | | |
| Region 13 | saccharide | 3,413,794 | 3,457,881 | pseudopyronine A/pseudopyronine B | Other:Fatty acid | 0,43 |
| Region 14 | RiPP-like | 3,546,262 | 3,558,460 | | | |

| | | | | | | |
|---|---|---|---|---|---|---|
| Region 15 | halogenated | 3,707,967 | 3,728,563 | | | |
| Region 16 | NRPS | 3,745,019 | 3,820,558 | lokisin | NRP | 0,85 |
| Region 17 | betalactone | 4,129,201 | 4,152,461 | fengycin | NRP | 0,13 |
| Region 18 | saccharide | 4,398,129 | 4,434,661 | capsular polysaccharide | Saccharide | 0,12 |
| Region 19 | NRPS | 4,518,212 | 4,571,208 | Pf-5 pyoverdine | NRP | 0,18 |
| Region 20 | fatty_acid,saccharide | 4,639,564 | 4,684,052 | O-antigen | Saccharide | 0,35 |
| Region 21 | NAGGN | 4,711,438 | 4,726,327 | | | |
| Region 22 | fatty_acid | 4,759,672 | 4,777,619 | | | |
| Region 23 | fatty_acid | 4,821,225 | 4,842,445 | mevalagmapeptide A/mevalagmapeptide B/mevalagmapeptide C/mevalagmapeptide D | NRP | 0,04 |
| Region 24 | saccharide | 5,287,032 | 5,322,048 | gamexpeptide A/gamexpeptide B/gamexpeptide E/luminmide B/luminmide D/luminmide E/luminmide F/luminmide G | NRP | 0,18 |
| Region 25 | saccharide | 5,373,804 | 5,397,007 | | | |
| Region 26 | saccharide | 5,766,772 | 5,787,533 | | | |

| | | | | | | |
|---|---|---|---|---|---|---|
| Region 27 | RRE-containing | 5,916,908 | 5,937,183 | lankacidin C | NRP+Polyketide | 0,13 |
| Region 28 | saccharide | 6,483,787 | 6,516,211 | S-layer glycan | Saccharide | 0,12 |

**Supplementary Table 5.** PleB sequences from other reported members of the amphisin family of LP.

| **accession** | **protein** | **organism** | **family** |
|---|---|---|---|
| ABW17379.1 | MacB | [Pseudomonas putida] | amphisin |
| MBY8934714.1 | MacB | [Pseudomonas fluorescens] | amphisin |
| WP_064118557.1 | MacB | [Pseudomonas fluorescens] | amphisin |
| BAF40423.1 | MacB | [Pseudomonas sp. MIS38] | amphisin |
| QDF82252.1 | MacB | [Pseudomonas sp.] | amphisin |
| QNL34620.1 | MacB | [Pseudomonas sp.] | amphisin |
| WP_175554031.1 | MacB | [Pseudomonas] | amphisin |
| MacB_R5_CFBP2392 | MacB | [Pseudomonas fluorescens] | |

## 3 Supplementary Figures

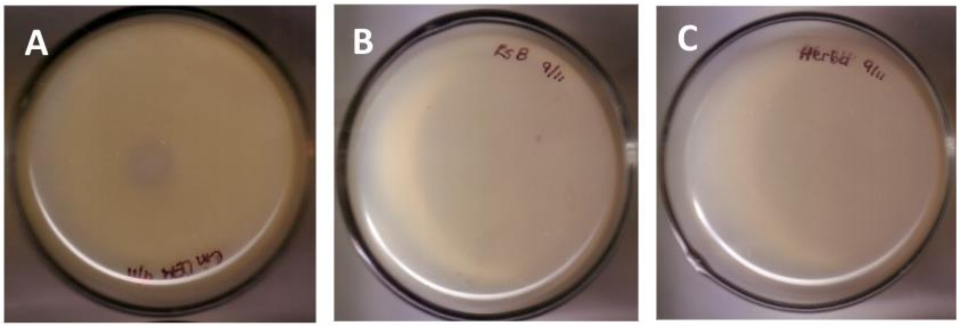


**Supplementary Figure 1.** Antibacterial activity spectrum of *Pseudomonas fluorescens* CFBP2392. In vitro antagonism assay against other plant pathogenic bacteria reveals the strain has inhibitory

activity against A) Gram positive *Clavibacter michiganensis* but not Gram negative B) *Ralstonia solanacearum* Rs8 or C) *Herbaspirillum seropedicae* Z69.

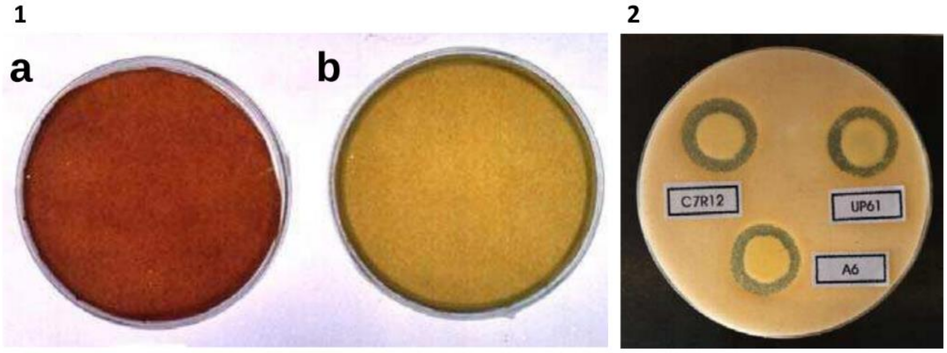


**Supplementary Figure 2.** 1) HCN production by (a) *P. fluorescens* CFBP2392 (b) negative control. 2) Exoprotease production by *P. fluorescens* CFBP2392 (A6). The figure shows protease production of two other *P. fluorescens* strains C7R12 and UP61.

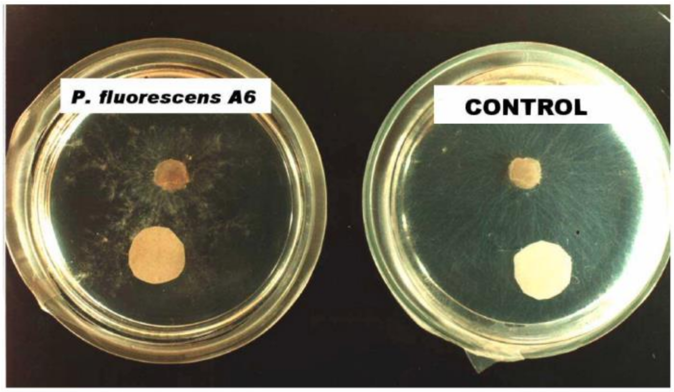


**Supplementary Figure 3.** Diffusion bioassay to evaluate the antifungal activity of the organic extract of a *P. fluorescens* CFBP2392 (A6) culture (left). In the control plate (right) the paper disk was loaded with methanol.

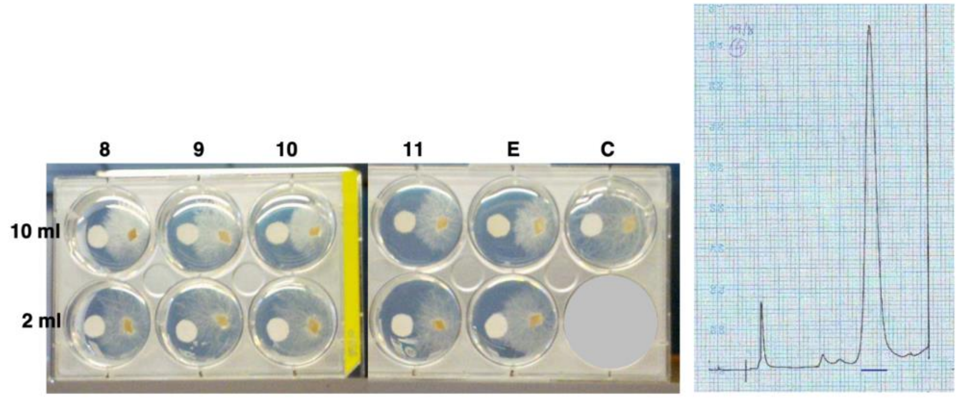


**Supplementary Figure 4.** Bioassay to evaluate the antifungal activity of the fractions obtained by adsorption chromatography (left). The figure shows only the result with fractions 8, 9, 10 and 11. The other fractions did not show activity under the conditions used. E: unfractionated organic extract; C: control with acetone (solvent used to dissolve the fractions). The amount from 10 ml or 2 ml of the original culture was loaded into the discs of the wells located in the upper or lower part of the figure, respectively. HPLC elution profile of fraction S3 obtained by size exclusion chromatography (right). The mobile phase consisted of a linear gradient of 0.07% acetonitrile-TFA in 0.1% $H_2O$-TFA in the following steps: 0% from 0 to 10 min, 0-12% from 10 to 45 min, 12-42% from 45 to 55 min and 42-

100% from 55 to 65 min. The flow used was 2 ml/min. The collected peak is marked with a line at the base.

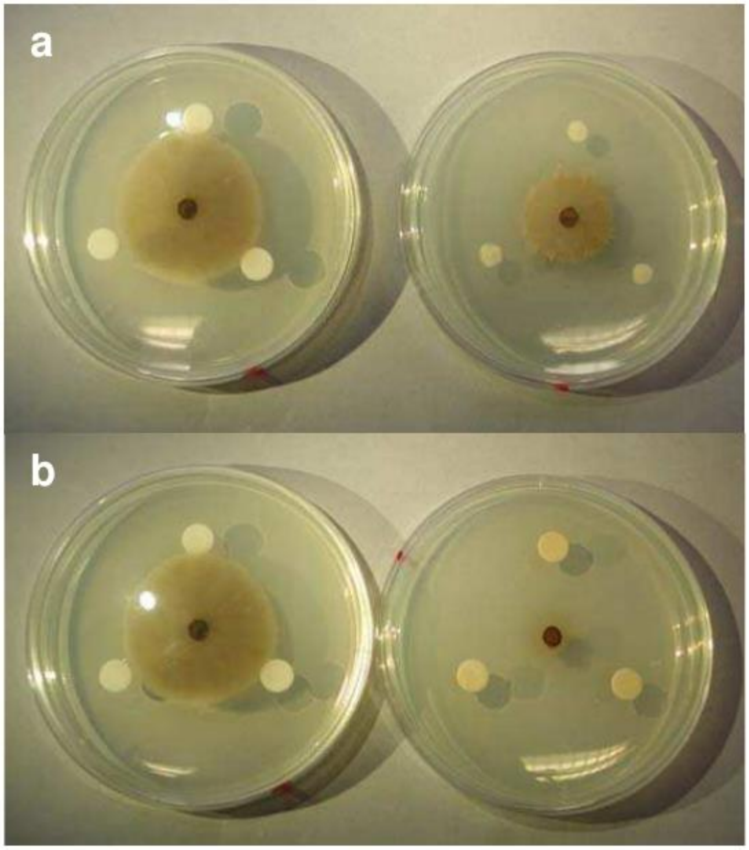


**Supplementary Figure 5.** Inhibition of *R. solani* RB1 by *P. fluorescens* CFBP2392 (a) or by the partially purified compound (CPP) diluted 1/2 (b). The plates to the left of each photo are the controls.

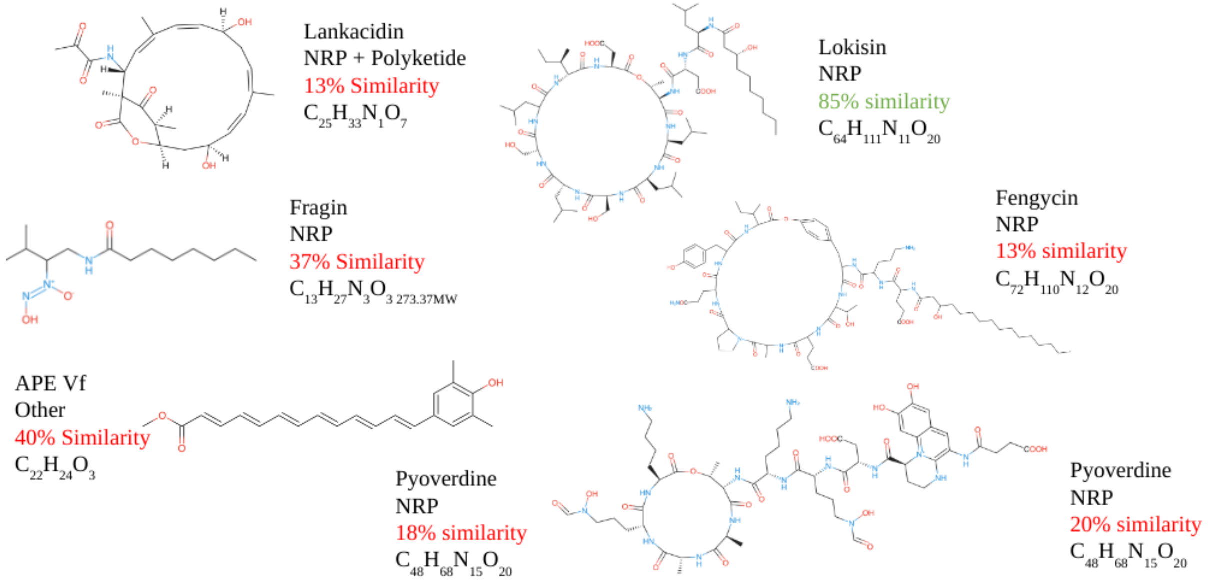


**Supplementary Figure 5.** Most similar compounds predicted for seven of the ten BGC regions in *P. fluorescens* CFBP2392. Six compounds are predicted to have less than 40% similarity with other reported compounds. Lokisin is predicted to have the highest similarity.

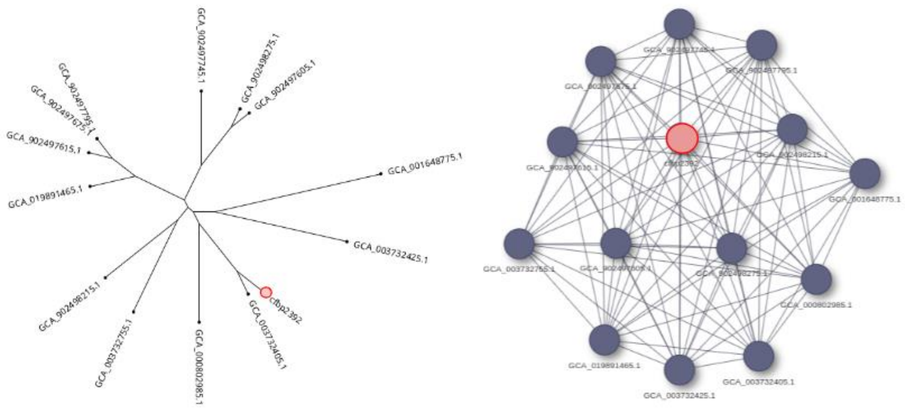

**Supplementary Figure 6.** Phylogeny of 14 selected genomes of *P. fluorescens* based on the predicted core genes (n = 4560) (left). Network visualization analysis of the selected genomes based on the presence/absence of known metabolite clusters.

## 4 Supplementary References

Bakker, A. W., and Schippers, B. (1987). Microbial cyanide production in the rhizosphere in relation to potato yield reduction and Pseudomonas SPP-mediated plant growth-stimulation. Soil Biol. Biochem. 19, 451–457. doi: 10.1016/0038-0717(87)90037-X.

Dunne, C., Crowley, J. J., Moënne-Loccoz, Y., Dowling, D. N., Bruijn,  s, and O'Gara, F. (1997). Biological control of Pythium ultimum by Stenotrophomonas maltophilia W81 is mediated by an extracellular proteolytic activity. Microbiology 143, 3921–3931. doi: 10.1099/00221287-143-12-3921.
